# Detection of circulating sarcoma tumor cells using a microfluidic chip-type cell sorter

**DOI:** 10.1038/s41598-019-56377-z

**Published:** 2019-12-27

**Authors:** Nobuhiko Hasegawa, Ikuko Takeda Nakamura, Toshihide Ueno, Shinya Kojima, Masahito Kawazu, Keisuke Akaike, Taketo Okubo, Tatsuya Takagi, Yoshiyuki Suehara, Takuo Hayashi, Tsuyoshi Saito, Kazuo Kaneko, Hiroyuki Mano, Shinji Kohsaka

**Affiliations:** 1 Division of Cellular Signaling, National Cancer Center Research Institute, 5-1-1 Tsukiji, Chuo-ku, Tokyo 104-0045 Japan; 2 Department of Orthopedic Surgery, Juntendo University, Graduate School of Medicine, Tokyo, 113-8431 Japan; 3 Department of Respiratory Medicine, Juntendo University, Graduate School of Medicine, Tokyo, 113-8431 Japan; 4 Department of Human Pathology, Juntendo University, Graduate School of Medicine, Tokyo, 113-8431 Japan

**Keywords:** Cancer genomics, Sarcoma

## Abstract

Analyses of circulating tumor cells have been shown to be effective for the detection of cancer relapse and prognosis prediction. However, research regarding its utility in sarcoma remains scarce. In this study, the microfluidic chip-type cell sorter On-chip Sort was used to construct a system for detecting circulating sarcoma cells (CSCs). A pilot study using normal fibroblast or sarcoma cell lines was designed to establish a reliable protocol to separate CSCs by On-chip Sort. A single CSC was separated and recovered from 10 ml of whole blood from a patient with locally advanced myxofibrosarcoma. The nonsynonymous mutation for *KMT2B* p.Ile2602Val identified in the formalin-fixed paraffin-embedded tumor sample was also confirmed in the CSC. Use of the developed protocol may allow CSCs to become an early predictor for metastasis and recurrence of sarcoma. Further, it may aid in optimizing post-operative therapies for patients without metastasis.

## Introduction

Soft tissue sarcoma (STS) is a rare malignant tumor of various histological types that arises from bones and soft tissues^1^. High-grade STS is managed using surgical resection, radiation, and chemotherapy^2^. Despite these multidisciplinary therapies, metastasis remains a critical issue^3^. Indeed, the 5-year overall survival (OS) rate of advanced STS is only 30%, while that of localized STS is 70%^4^.

Although newer molecular-targeting drugs, such as imatinib, pazopanib, and olaratumab, have been approved for the treatment of STS^5,6^, the response rates are not very high^7^. Therefore, doxorubicin, which was developed about 30 years ago, is still used as the first-line treatment. However, doxorubicin treatment is often associated with severe adverse effects, such as neutropenia and a neutropenic fever^8^. It is therefore controversial to administer chemotherapy to patients without metastasis.

Detecting early recurrence and metastasis is essential to providing chemotherapy in a timely manner. Radiographic imaging, including computed tomography (CT) and magnetic resonance imaging (MRI), are routinely used for follow-up after STS surgery^9^. Metastasic loci often spread throughout the body before such imaging systems detect metastatic regions. Therefore, high-sensitivity methods for detecting recurrence and metastasis are needed.

Recently, several biomarkers have been established for predicting metastasis and assessing tumor activity. These include circulating tumor cells (CTCs), cell-free DNA (cfDNA), and circulating microRNAs^10,11^. The number of CTCs is a particularly strong prognostic factor. CTCs can be detected in the peripheral blood of patients with metastatic and localized tumors^12^. The detection of CTCs has been performed across various tumor types, especially in epithelial carcinomas, such as lung cancer, breast cancer, and prostate cancer^13,14^. However, research regarding its utility in sarcoma remains scarce^15^.

The CELLSEARCH System (Veridex, NJ, USA) is the only assay for CTC enumeration approved by the U.S. Food and Drug Administration^16^. In this system, cell-surface expression of epithelial cell adhesion molecule‐1 (EpCAM) is used to detect CTCs. EpCAM is a standard marker of epithelial tumors. However, EpCAM is not generally expressed in STS, making an alternative method necessary to collect circulating sarcoma cells (CSCs). Although the confirmation of fusion genes in CSCs have been examined^17^, detection of somatic mutations, such as single nucleotide variants or small insertion/deletion, has not yet been performed.

On-chip Sort (On-chip Biotechnologies, Tokyo, Japan) is a cell sorter that uses a disposable microfluidics chip and includes a collection reservoir to store the target cells^18,19^. The principle of On-chip Sort comprises flow cytometry using the FISHMAN-R system^20^. Unlike commonly used cell sorters, this device can sort repeatedly without damaging cells. When a highly concentrated sample is analyzed, the target is reliably recovered without loss of target cells, but with a significant number of nonspecific cells. However, the target recovery rate improved when the cell concentration was sufficiently reduced after repeated sorting to accurately differentiate the target cells. Use of this device for the collection of CTCs has been validated for lung cancer^21,22^.

The current study aimed to establish a protocol for CSC collection and molecular profiling using On-chip Sort. The Todai OncoPanel (TOP), a multi-gene panel test that examines 464 genes^23^, was used to confirm that CSCs harbored somatic mutations that were identical to the original tumor.

## Results

### Protocol for CSC enrichment, enumeration, sorting, and sequencing

**Figure 1:**
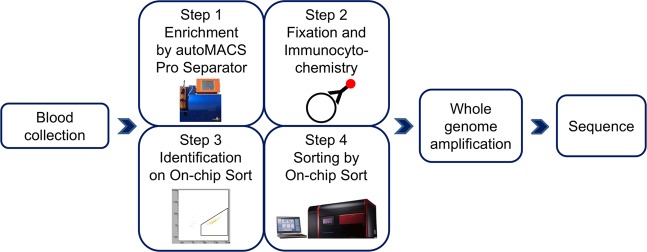
Protocol for CSC enrichment, enumeration, sorting, and sequencing. An overview of CSC analysis in this study. The whole blood obtained from a sarcoma patient was enriched using autoMACS Pro Separator (Step 1). CSCs were fixed and stained with antibodies specific for sarcoma or WBCs (Step 2). CSC enumeration (Step 3) and sorting (Step 4) were carried out using the On‐chip Sort system. Genome sequencing was performed after WGA of CSCs. The permission for the usage of image of On-chip Sort was obtained from On-chip Biotechnologies.

An overview of the CSC analyses used in this study is shown Fig. 1. Blood cell depletion was carried out by autoMACS Pro Separator using CD45 MicroBeads and CD235a MicroBeads. The CD45 antigen is expressed in all cells of hematopoietic origin except erythrocytes, platelets, and their precursor cells. The CD235a antigen (glycophorin A), a single-pass transmembrane glycoprotein, is expressed in mature erythrocytes and erythroid precursor cells.

The remaining cells were fixed and stained with antibodies specific for sarcoma, vimentin, or white blood cells (WBCs), such as CD45 and CD14. The CD14 antigen, a component of the LPS receptor complex, is strongly expressed in most monocytes and macrophages and weakly expressed in neutrophils and some myeloid dendritic cells. Conversely, vimentin is expressed in a wide range of cell types. These include stromal cells, fibroblasts, endothelial cells, and neuronal precursor cells. It was hypothesized that CD45/14 (–)/vimentin (+) fractions in peripheral blood may specifically contain CSCs.

CSC enumeration and sorting were carried out using the On‐chip Sort system (On-chip Biotechnologies). Genome sequencing was performed after whole-genome amplification (WGA) of CSCs (Fig. 1).

**Figure 2:**
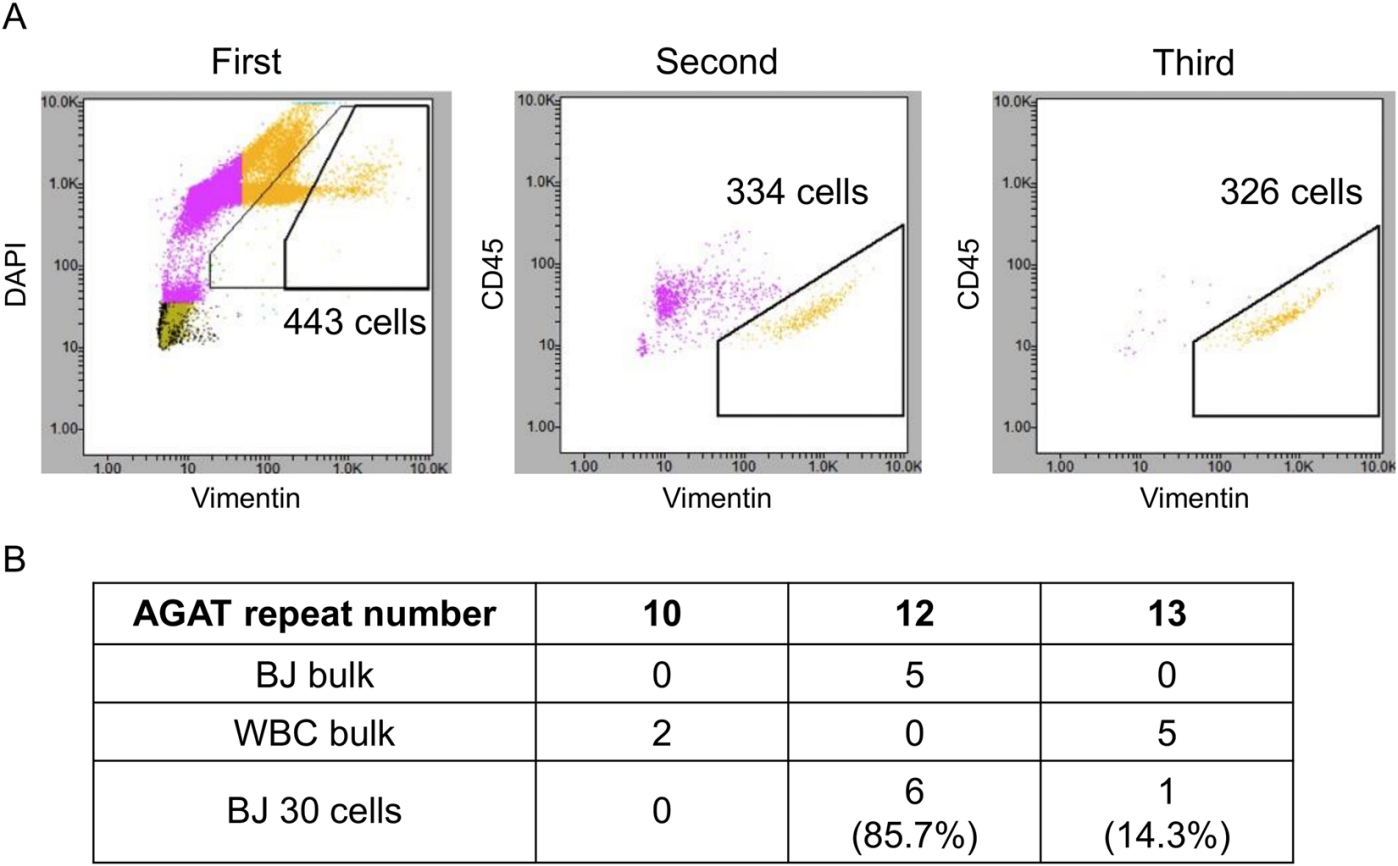
Protocol establishment and validation using a normal human fibroblast cell line. (**A**) A total of 500 BJ cells were spiked into WBCs from healthy volunteers (3.0 × 10^5^ cells). The samples were then stained with DAPI, CD45, CD14, and vimentin and sorted three times using On-chip Sort. The first gate was DAPI (+)/vimentin (+). The second and third gates were CD45 (−)/vimentin (+), resulting in 326 sorted cells. **(B)** The STRs of the WGA sample were determined to investigate the composition of the sorted cells. BJ bulk: DNA extracted from bulk BJ cells, WBC bulk: DNA extracted from bulk WBCs, BJ 30 cells: WGA DNA of sorted BJ cells using On-chip Sort.

A pilot study using the human fibroblast cell line BJ was designed as follows. Five hundred BJ cells were spiked in 3.0 × 10^5^ of WBCs. The sample was then fixed and stained with anti-CD45 mouse monoclonal antibody (mAb) conjugated to allophycocyanin (APC), anti-CD14 mouse mAb conjugated to APC, 4′,6-Diamidino-2-phenylindole dihydrochloride (DAPI), and anti-vimentin Alexa Flour 488-conjugated rabbit mAb. We intended to enrich spiked BJ cells (skin fibroblasts) by reducing WBCs (CD45-positive), macrophages, and monocytes (CD14-positive). To completely remove macrophages and monocytes, we utilized an APC-conjugated anti-CD45 Ab combined with an anti-CD14 Ab. A CD45/14 (−)/vimentin (+) fraction from the sample was purified with the On‐chip Sort system. Sorting was repeated three times to enrich CSCs. After purification, a total of 326 cells were gated as CD45 (−)/vimentin (+), and aliquoted into 10 tubes (containing ~30 cells per tube) (Fig. 2A). One of those tubes was subjected to WGA.

The short tandem repeats (STRs) determined in the WGA sample were used to investigate the composition of the sorted cells. The STR locus D5S818 of the BJ genome contains 12 repetitions of “AGAT” in both alleles, whereas WBCs have 10 repetitions in one allele and 13 repetitions in the other. As shown in Fig. 2B, Sanger sequencing of the polymerase chain reaction (PCR) products for the D5S818 locus revealed that 85.7% of the sorted fraction were identified as BJ cells.

### WBC removal using autoMACS Pro Separator

**Figure 3:**
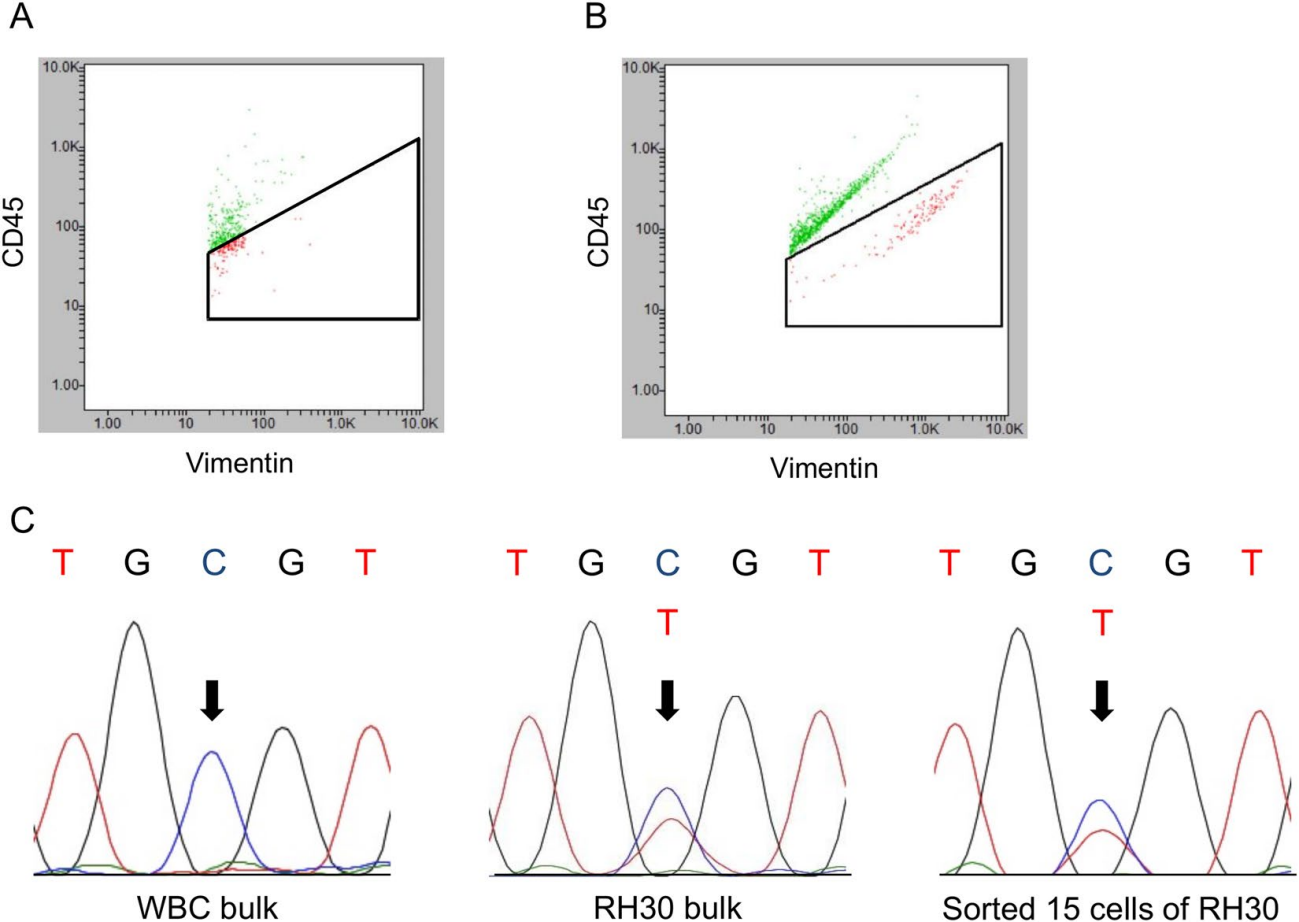
WBC removal using autoMACS Pro Separator. **(A)** The sample underwent RBC lysis without WBC depletion before running On-chip Sort. RH30 cells were not well-separated from WBCs. **(B)** After using autoMACS Pro Separator to remove WBCs from 2 ml of whole blood, RH30 cells and WBC were clearly separated by On-chip Sort. **(C)** Sanger sequence was performed to confirm the *TP53* c.817C > T mutation (p.Arg 273 Cys), a known mutation in the RH30 cell line. The electropherograms of bulk WBCs (left panel), bulk RH30 cells (middle panel), and sorted 15 cells of RH30 (right panel) are shown.

The necessity of WBC depletion from the whole blood sample before running On-chip Sort was next explored. Approximately 50 cells of the rhabdomyosarcoma cell line RH30 were spiked into 2 ml of whole blood collected from a healthy volunteer. One sample underwent red blood cell (RBC) lysis without a negative selection for WBCs. The sample was then fixed, stained, and run through On-chip Sort. However, RH30 cells were not well-separated from the WBC group (Fig. 3A). In contrast, RH30 cells were clearly identified after removing WBCs from the sample using autoMACS Pro Separator (Fig. 3B).

Forty-seven cells gated as CD45 (−)/vimentin (+) were collected and divided into three tubes. One of those tubes was subjected to WGA. Sanger sequencing was performed to confirm the *TP53* c.817C > T DNA mutation (protein mutation: p.Arg273 Cys) in the amplified DNA, which is known to be present in the RH30 genome. The electrophoretogram of the sorted cells was the same as that of bulk RH30 cells (Fig. 3C). Thus, depleting WBCs before loading the sample onto On-chip Sort improved the separation accuracy for sorting spiked sarcoma cells from whole blood samples.

### Method for gating CSCs using WBCs from the same patient on On-chip Sort

**Figure 4:**
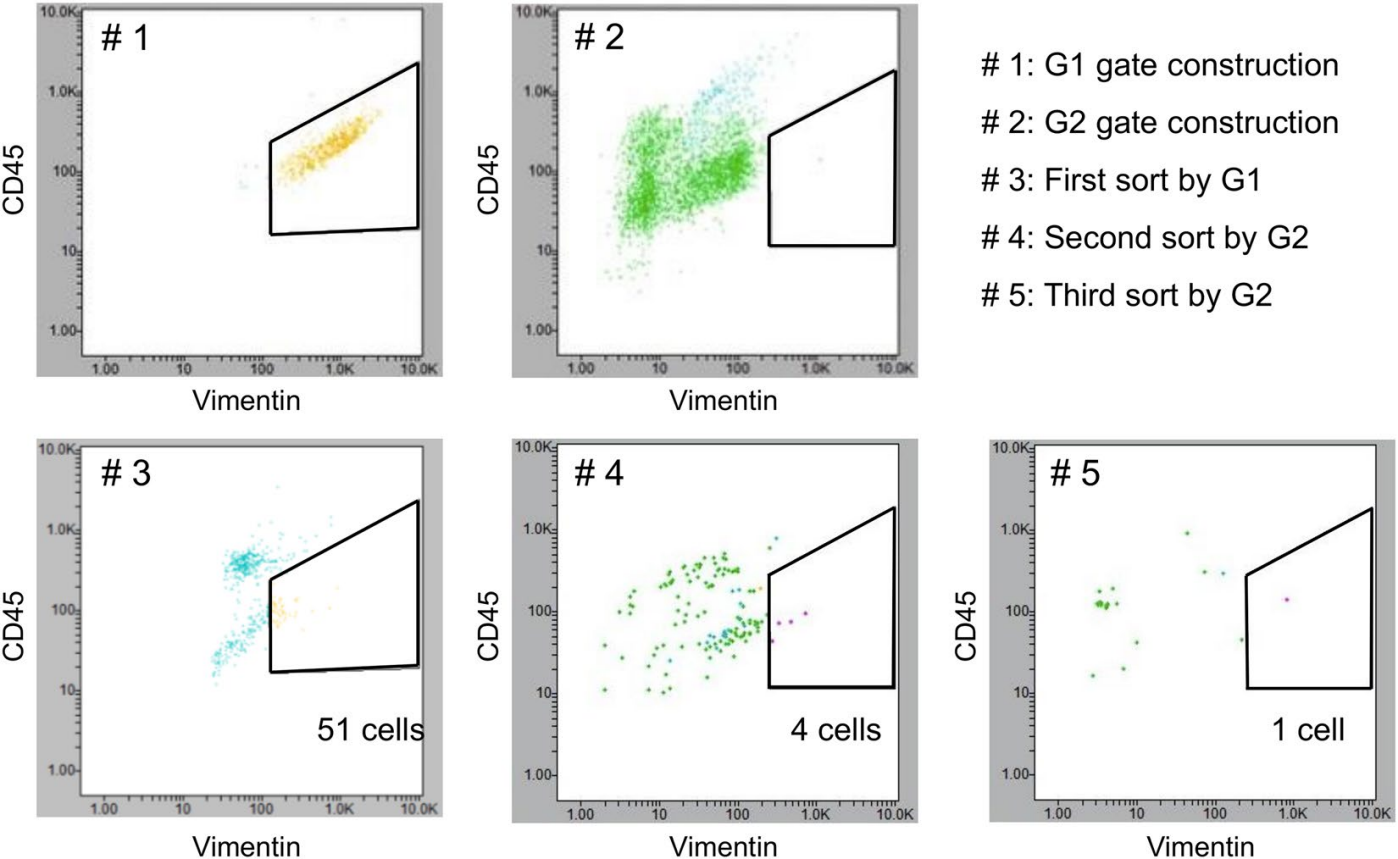
CSC sorting in a 60-year-old man with myxofibrosarcoma. A CSC-specific gate including ≥90% of RH30 cells (G1) was created using simultaneously prepared RH30 cells as a vimentin (+) control. A gate that included no WBCs from the patient (G2) was created using the patient’s WBCs as a vimentin (−) control. WBCs were separated in advance from 10 ml of whole blood by autoMACS Pro Separator. The first round of sorting using the G1 gate resulted in 51 cells. The second and the third rounds using the G2 gate resulted in four cells and one cell, respectively.

To construct an appropriate gate for vimentin, RH30 was always analyzed as a positive control cell line in parallel to the specimens of interest. The CSC gates for the flow cytometer were set to contain ≥90% of RH30 cells (G1) and no WBCs of the patient (G2). WBCs were separated in advance from 10 ml of whole blood using autoMACS Pro Separator. The first round of sorting was performed using the G1 gate, and the second and the third rounds were performed using the G2 gate (Fig. 4).

### Detection of CSCs in a myxofibrosarcoma patient

A 60-year-old man with myxofibrosarcoma (histological grade: Fédération Nationale des Centers de Lutte Contre le Cancer grade 3, disease stage: American Joint Committee on Cancer/Union for International Cancer Control tumor-node-metastasis stage III) underwent preoperative chemotherapy, followed by surgical excision and post-operative chemotherapy. A blood sample was collected from the patient before the preoperative chemotherapy, 3 months post-, and 9 months post-surgery. No clear metastases were observed at any point during blood sampling (Fig. S1).

Erythrocytes and WBCs were removed from the collected blood specimens. The samples were then fixed and stained with antibodies for CD45, CD14, DAPI, and vimentin. CSC enumeration and sorting was performed using On-chip Sort. A single CSC was sorted at 3 months after surgery (Fig. 4), while no CSCs were detected before treatment or at 9 months after surgery (Fig. S2).

### Formalin-fixed paraffin-embedded (FFPE) tumor tissue, normal tissue, CSC, and cfDNA sequencing

To determine whether alterations in the genomes of primary tumor cells can be detected in CSCs and cfDNA, we analyzed genomic alterations in single CSCs and cfDNAs three months after surgery and compared them with those found in the primary tumor FFPE sample. Genomic DNA of tumor or normal tissues isolated from the FFPE and peripheral blood specimen of the patient were subjected to extensive sequencing for cancer-related genes using the TOP panel^23^. A subsequent bioinformatics analysis revealed cancer-associated somatic mutations, including *KMT2B* c.7804A > G DNA (protein: p.Ile2602Val) and *MGA* c.3628C > G DNA (protein: p.Arg1210Gly) with allele frequencies of 13.06% and 6.12%, respectively (Table 1).

**Table 1:** Identification of somatic mutation in CSC.

Gene name	Sample	HGVS c.	HGVS p.	Depth	Variant read	VAF (%)
*KMT2B*	FFPE	c.7804 A > G	p.Ile2602Val	1692	221	13.1
	CSC	c.7804 A > G	p.Ile2602Val	122760	42845	34.9
	cfDNA	c.7804 A > G	p.Ile2602Val	570143	1028	0.18
		c.7804 A > C	p.Ile2602Leu		66	0.011
		c.7804 A > T	p.Ile2602Phe		87	0.015
	cfDNA (GCTB-1)	c.7804 A > G	p.Ile2602Val	694481	1343	0.19
		c.7804 A > C	p.Ile2602Leu		94	0.013
		c.7804 A > T	p.Ile2602Phe		64	0.0092
	cfDNA (GCTB-2)	c.7804 A > G	p.Ile2602Val	692495	1412	0.20
		c.7804 A > C	p.Ile2602Leu		76	0.0098
		c.7804 A > T	p.Ile2602Phe		63	0.0090
*MGA*	FFPE	c.3628 C > G	p.Arg1210Gly	588	36	6.12
	CSC, cfDNA	c.3628 C > G	p.Arg1210Gly	NA	NA	NA

HGVS, Human Genome Variation Society; c., coding DNA sequence; p., protein sequence; VAF, variant allele frequency.

FFPE, formalin-fixed paraffin-embedded; CSC, circulating sarcoma cell; cfDNA, cell-free DNA; GCTB, giant cell tumor of bone; NA, not applied.

Targeted deep sequencing of *KMT2B* was performed in the CSC DNA and cfDNA obtained 3 months after surgery. Importantly, the *KMT2B* c.7804A > G mutation was confirmed to be present in the CSC [variant allele frequency (VAF) of 34.9%], whereas *KMT2B* c.7804A > G, c.7804A > C, and c.7804A > T were also observed in the cfDNA at 0.18%, 0.011%, and 0.015%, respectively (Table 1). To verify that the *KMT2B* mutations observed in cfDNA at low VAFs were true positive mutations, targeted deep sequencing was performed in two giant cell tumor of bone (GCTB) specimens that did not have *KMT2B* c.7804A > G in the original tumors. The *KMT2B* c.7804A > G mutation was also found in the GCTB tumors at VAFs of 0.19% and 0.20% (Table 1). Therefore, the *KMT2B* mutations found at low VAFs were likely to be sequence errors.

## Discussion

The current study established a new protocol for CSC detection using a microfluidic chip-type cell sorter, the On-chip Sort. Several reports have described detection of CSCs using different devices, such as a size-exclusion microfiltration system^24^ and flow cytometry^25,26^ with specific markers. However, the utility of size-exclusion is limited by the lack of a histology-specific marker. Flow cytometry is limited due to the heterogeneous expression of target markers, which may affect the detection sensitivity. Even specific markers, such as CD99 for Ewing’s sarcoma, show background expression on normal hematopoietic cells^27^.

As there were no standard markers suitable for CSC isolation, vimentin was used to target mesenchymal cells^28^. However, WBCs are also known to express vimentin to some extent. Thus, the number of WBCs was first reduced using a negative selection with the autoMACS Pro Separator system. Then, the CD45 (−)/vimentin (+) fractions were separated using On-chip Sort. Simultaneous analyses of RH30 as a vimentin (+) control and WBCs as a negative control from the same patient allowed for precise gating of the target fraction. Theoretically, the current protocol may be applicable for the isolation of any type of CSCs.

A previous report used real-time PCR to confirm the fusion genes of CSCs^29^. Although fusion genes are found in about one-third of sarcomas, no specific genetic abnormalities can be identified in the remaining two-thirds of sarcomas, including myxofibrosarcoma. Somatic mutations identified by the TOP panel can be utilized to confirm any type of CSCs. Reports on sarcoma cfDNAs have been published^30–32^, and efforts have been made to identify sarcoma mutations in cfDNA by improving sequencing techniques^33^. However, cfDNA remains difficult to detect in pre-metastatic cases. The current study showed that, in contrast to the CSC analysis, cfDNA was unable to detect the somatic KMT2B mutation found in the sarcoma. MicroRNAs have also attracted interest in the study of STS^34,35^, although their utility in clinical practice has not been demonstrated. By improving the sorting method, CSCs can be identified in patients before metastasis. These findings suggested that CSCs might be an early predictor of metastasis and recurrence in sarcoma and could help inform decisions concerning post-operative radiation therapy and chemotherapy for patients without metastasis. Further studies are needed to compare the limit of detection between CSCs and cfDNA analyses.

Presence of CTCs (three cells or more) was shown to predict shorter progression-free survival/OS for colon cancer patients^36^. While one CSC was detected at 3 months after surgery, the patient in the current study did not demonstrate any signs of metastases after 9 months of surgery. This may suggest that a few remaining CSCs are insufficient to colonize any metastatic sites. Quantitative evaluation of CSCs with the current protocol might help to stratify the prognosis of patients with few CSCs. The accuracy of separating CSCs can be further improved by adding other selection windows, such as CD99 for Ewing sarcoma or MDM2 for liposarcoma. The clinical utility of the current improved protocols must be tested in larger cohorts.

One of the limitations of this study is the sample preparation for the pilot study. The 65% recovery in this pilot experiment using the BJ cell line was relatively lower than that of the other platforms because the number of manually prepared BJ cells used for the spike was not 500. The other pilot experiment using the RH30 cell line shows that the isolation efficiency was sufficiently high (~90%). To calculate the accuracy of enrichment, we should use a flow cytometer to acquire an exact cell number for the spike experiment.

Overall, the accuracy in CSC collection and enumeration was markedly improved using the On-chip Sort system. The protocol suggested in this study may prove to be a fundamental tool for CSC collection. Future work may apply this protocol to laboratory testing for monitoring the recurrence and molecular profile of tumors, which will improve patient prognosis and maximize opportunities for treatment.

## Methods

### Cell preparation

BJ cells and RH30 cells (ATCC, Manassas, VA, USA) were cultured in EMEM and RPMI-1640 mediums, respectively. Media contained 10% fetal bovine serum and 1% penicillin-streptomycin solution. Cells were collected when they reached 70–80% confluence.

### Patients and tumor samples

This project was approved by the ethics review board of Juntendo University Hospital (No. 2018169). Written informed consent was obtained from the patients whose samples were analyzed in the present study. Samples were collected from a myxofibrosarcoma patient who received chemotherapy and two GCTB patients at the Department of Orthopedic Surgery in Juntendo University Hospital. A 10 ml sample of whole blood and FFPE were collected at the time of diagnosis. In the myxofibrosarcoma patient, blood samples were collected at three points: before treatment was started, 3 months post-, and 9 months post-surgery. In addition, whole blood was collected from healthy participants. All procedures performed in studies involving human participants were in accordance with the ethical standards of the institutional research committee and with the 1964 Helsinki declaration and its later amendments or comparable ethical standards.

### Immunomagnetic enrichment

Blood samples (10 ml) were collected with blood collection tubes (EDTA-2K) and used within 3 h. Whole blood was separated into two 15 mL Conical Centrifuge Tubes (Thermo Fisher Scientific, Waltham, MA, USA) and 5 ml of autoMACS Pro Separator Running Buffer was added to each sample. The samples were then centrifuged at 400 *g* for 10 min to separate the plasma without damaging the buffy coat. CD45 Micro Beads were added to each 5 ml sample at a concentration ratio of 1:20. The sample was then stirred by MACSmix Tube Rotator (Miltenyi Biotec, Bergisch Gladbach, Germany) for 15 min. After stirring, Running Buffer was added to bring the sample up to 13 ml to flush the CD45 MicroBeads attached to the tube wall. The samples were then centrifuged at 400* g* for 5 min to separate the plasma without damaging the buffy coat. The WBCs were then separated out using autoMACS Pro Separator. The negative fraction was mixed with 4 ml RBC Lysis Buffer (TONBO Bioscience, San Diego, CA, USA) and 28 ml UltraPure DNase/Rnase-Free Distilled Water (Thermo Fisher Scientific). The samples were then stirred using MACSmix Tube Rotator for 10 min. The samples were then centrifuged at 400* g* for 5 min to separate the supernatant. Running buffer (10 ml) was added to the samples, and the samples were centrifuged again at 400 *g* for 5 min and the supernatant was removed. Both samples were then mixed together and running buffer was added to bring the sample up to 500 µl. PE anti-human CD235a (Glycophorin A) antibody was also added at a concentration of 1:50. The sample was incubated for 10 min. Anti-PE MicroBeads (Miltenyi Biotec) were added to the sample at a concentration of 1:5 and incubated for 10 min. Running buffer was added up to 5 ml and the sample was then placed in autoMACS Pro Separator. The negative fraction was centrifuged at 300 *g* for 10 min and the supernatant was removed. Running buffer was added to bring the sample up to 500 µl, and the sample was centrifuged at 300 *g* for 10 min and the supernatant was removed. Running buffer was added up to 100 µl to load the sample onto On-chip Sort.

### CSCs enumeration and sorting using the On-chip Sort system

A 100-µl aliquot of the sample was fixed and stained. For fixing, 100 μl of Solution C (On-chip Biotechnologies) was added to the sample and incubated for 10 min. For cell permeabilization, 100 μl Solution D (On-chip Biotechnologies) was added to the sample and incubated for 20 min. Then 400 μl of On-chip T buffer was added to the sample before centrifuging at 400 *g* for 5 min. The supernatant was aspirated down to 100 μl. Sample staining was then performed using anti-CD45 mouse mAb conjugated to APC (1:40 dilution; Miltenyi Biotec), anti-CD14 mouse mAb (61D3) conjugated to APC (1:40 dilution; Cell Signaling Technology, Danvers, MA), Solution E-1 (DAPI) (1:100 dilution; On-chip Biotechnologies), anti-vimentin rabbit mAb (D21H3) conjugated to Alexa Flour 488 (1:50 dilution; Cell Signaling Technology), and Solution B for Fc receptor blocking (1:100 dilution; On-chip Biotechnologies). The sample was then incubated for 30 min. Enumeration and sorting of the cells were performed using On-chip Sort according to the manufacturer’s instructions^19^.

### WGA and PCR for genotyping

Cells sorted by On-chip Sort were transferred from the collection reservoir of the chip to a 200 μL PCR tube and centrifuged at 400 *g* for 10 min. Excess liquid was removed after centrifugation, leaving 2 μL of the liquid. WGA was performed using the SMARTer PicoPLEX WGA Kit (Takara Bio Inc., Shiga, Japan) following the manufacturer’s protocol. The amplified products were subjected to Sanger sequencing or next-generation sequencing (NGS).

### DNA extraction

Tumor tissue genomic DNA was extracted from FFPE samples using the Gene Read DNA FFPE Kit (Qiagen, Hilden, Germany). Genomic DNA of WBCs was extracted by QIAamp DNA Mini Kit (Qiagen). cfDNA was extracted from plasma using the QIAamp Circulating Nucleic Acid Kit (Qiagen). All kits were used according to the manufacturer’s protocols.

### Sanger sequencing

For capillary sequencing with a 3130xl Genetic Analyzer (Thermo Fisher Scientific), 10 ng of template DNA is used to amplify the STR locus of the D5S818 and *TP53* gene mutation (p.Arg 273 Cys) with GoTaq G2 Hot Start Green Master Mix (Promega, Madison, WI, USA) according to the manufacturer’s instructions. The following primer sets were used: the STR locus of D5S818, 5′-GGGTGATTTTCCTCTTTGGT-3′ (sense) and 5′-TGATTCCAATCATAGCCACA-3′ (antisense), and *TP53* gene mutation (p.Arg 273 Cys), 5′-GGGACAGGTAGGACCTGATTTCC-3′ (sense) and 5′-GTGGTGAGGCTCCCCTTTCTTG-3′ (antisense).

### Mutational profiling of primary tumor by TOP

The detailed protocol and the content of gene panel were described in previous paper^23^. In brief, gDNA (500 ng) of tumor FFPE and normal blood samples were subjected to target fragment enrichment using a SureSelectXT Custom kit (Agilent Technologies). Custom-made probes were designed to hybridize and capture the gDNA of the target genes listed by TOP. Massive parallel sequencing of the isolated fragments was performed using a HiSeq. 2500 (Illumina, San Diego, CA, USA).

### Targeted deep sequencing of CSCs and cfDNA

The library for deep sequencing was generated using the NEBNext Ultra DNA Library Prep Kit (New England Biolabs, Ipswich, MA, USA) according to the manufacturer’s instructions. WGA DNA of CSCs (100 ng) or cfDNA (100 ng) was subjected to PCR with the following primers for the *KMT2B* mutation (p.Ile2602Val): 5′-CCTGTTCTGTAAGCGCAACATCG -3′ (sense) and 5′-CTTGTCAGTCAACACCGAGCG-3′ (antisense). The PCR products were purified using AMPure beads (Beckman Coulter, Brea, CA, USA) for library preparation. The library quality was assessed using a Qubit 2.0 Fluorometer (Thermo Fisher Scientific) and the Agilent 2200 TapeStation System (Agilent, Foster City, CA, USA). The library was sequenced on MiSeq (Illumina) using a Reagent Micro Kit V2 (300-cycles) with the 150-bp paired-end option.

### NGS analysis

Target sequencing reads of gDNA from FFPE, blood, CSC, and cfDNA were independently aligned to the human reference genome (University of California Santa Cruz Genome Browser assembly ID: hg38) using Burrows-Wheeler Aligner (http://bio-bwa.sourceforge.net/) and Bowtie2 (http://bowtie-bio.sourceforge.net/bowtie2/index.shtml). For target sequencing using FFPE, PCR duplicate reads were removed using BamUtil (https://genome.sph.umich.edu/wiki/BamUtil). Somatic mutations of gDNA were identified using MuTect2 (GATK 4.0.11.0, https://software.broadinstitute.org/gatk/), VarScan2 (http://dkoboldt.github.io/varscan/) and an in-house pipeline. Mutations were discarded if: (i) the read depth was <100 or VAF was <0.01, (ii) they were supported by only one strand of the genome, or (iii) they were present in normal human genomes in either the 1000 Genomes Project dataset (http://www.internationalgenome.org/) or the in-house database. Gene mutations were annotated by SnpEff (http://snpeff.sourceforge.net/). Mutations of CSC and cfDNA were identified by an in-house method using SAMools (http://samtools.sourceforge.net/).

## Supplementary Information

Supplementary Information

Supplementary Information 2
