# Supplementary figures and images for: Detection of circulating sarcoma tumor cells using a microfluidic chip-type cell sorter
Source: Sci Rep. 2019 Dec 27;9:20047. doi: 10.1038/s41598-019-56377-z (PMC6934608; doi:10.1038/s41598-019-56377-z)

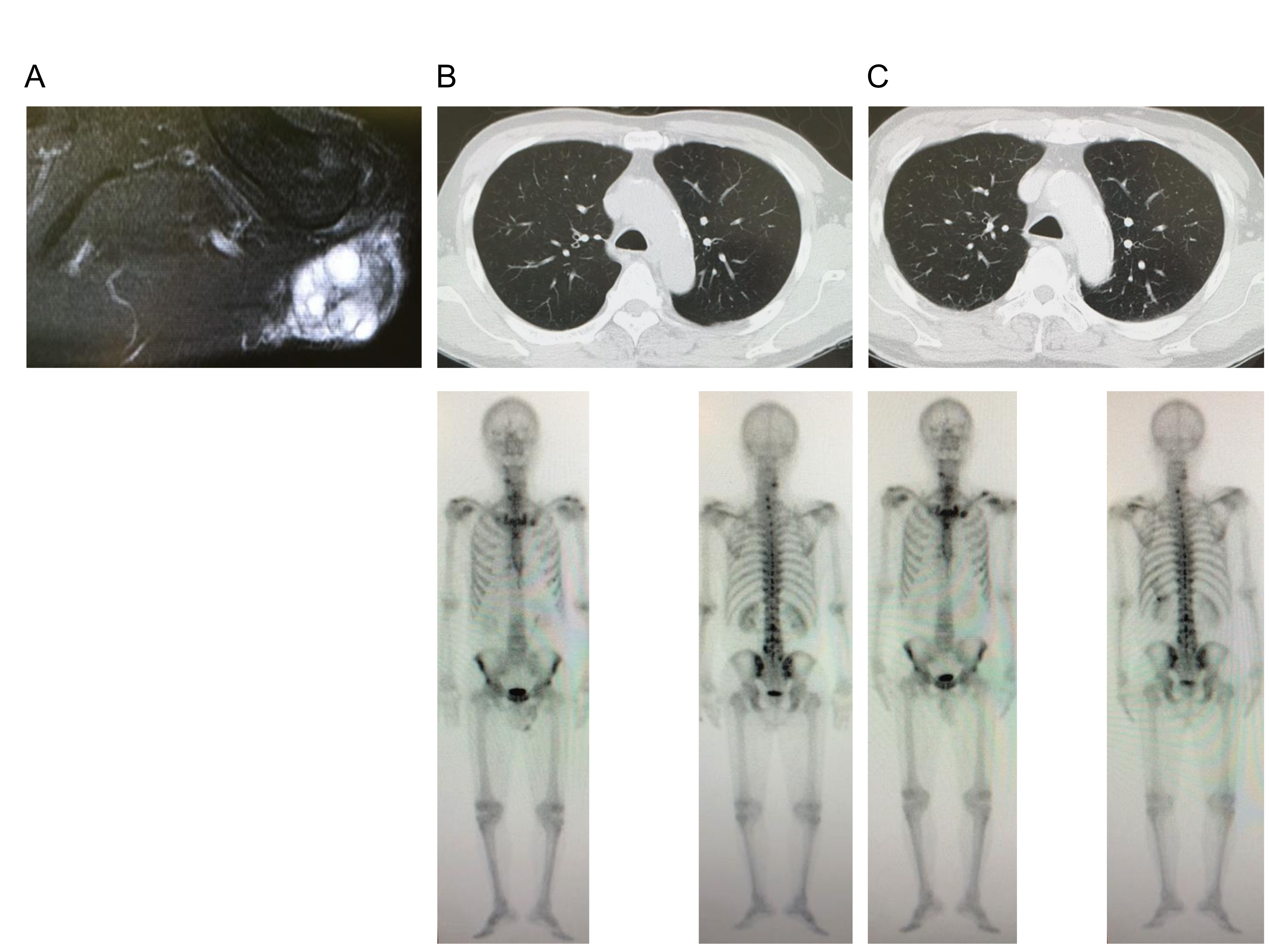

Supplement: Supplementary file 1 — Supplementary Information [file 41598_2019_56377_MOESM1_ESM.tif]

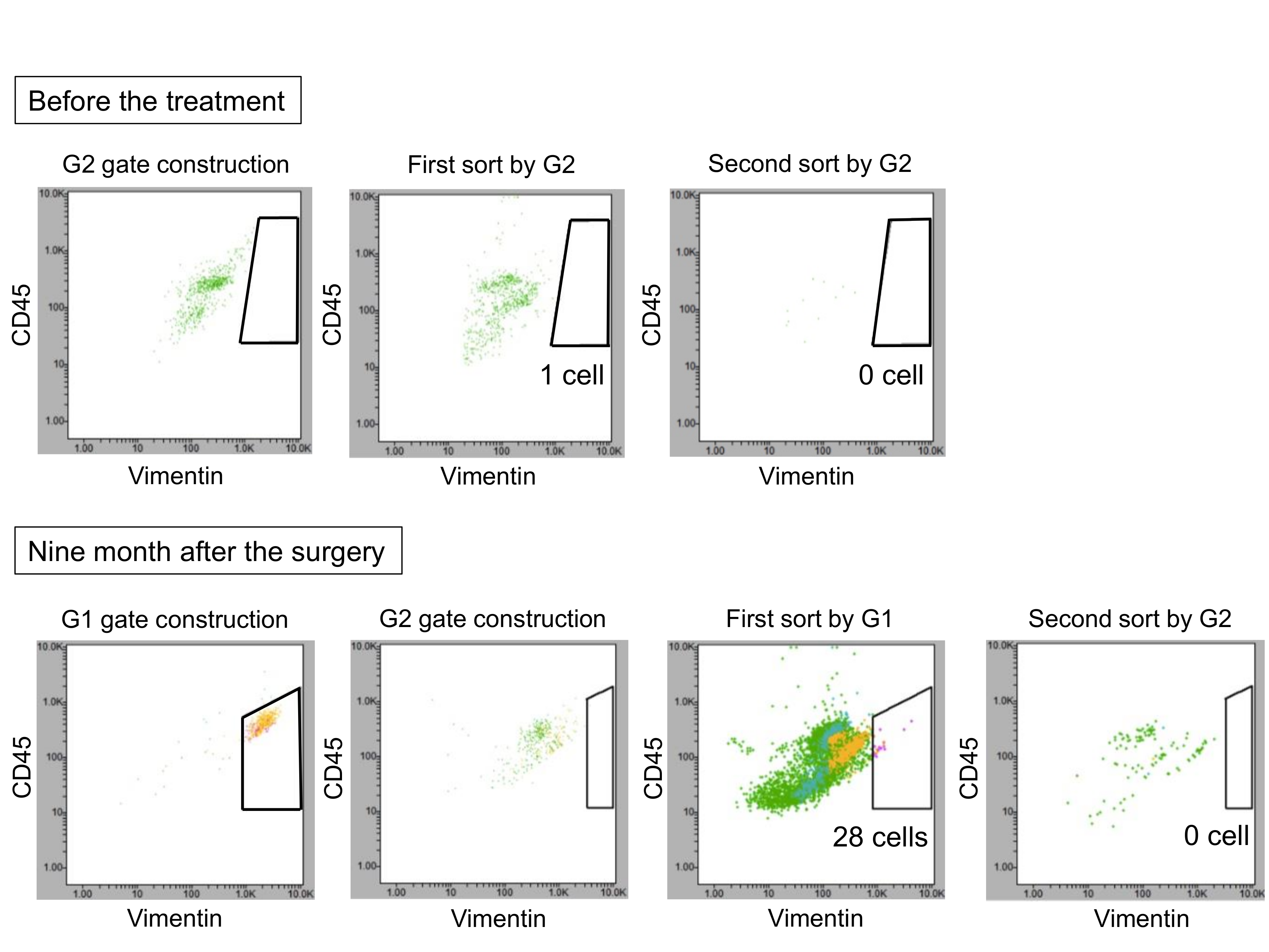

Supplement: Supplementary file 2 — Supplementary Information 2 [file 41598_2019_56377_MOESM2_ESM.tif]
